# Pyramidal system involvement in progressive supranuclear palsy – a clinicopathological correlation

**DOI:** 10.1186/s12883-019-1270-1

**Published:** 2019-03-20

**Authors:** Zuzana Stejskalova, Zdenek Rohan, Robert Rusina, Adam Tesar, Jaromir Kukal, Gabor G. Kovacs, Ales Bartos, Radoslav Matej

**Affiliations:** 1 Department of Pathology and Molecular Medicine Third Faculty of Medicine, Charles University and Thomayer Hospital, Videnska 800, 14059 Prague 4 – Krc, Czech Republic; 2 Department of Pathology, First Faculty of Medicine, Charles University and General University Hospital, Prague, Czech Republic; 3 Department of Pathology, Third Faculty of Medicine, Charles University and University Hospital Kralovske Vinohrady, Prague, Czech Republic; 4 Department of Neurology and Center of Clinical Neuroscience, First Faculty of Medicine, Charles University and General University Hospital, Prague, Czech Republic; 5 Department of Neurology Third Faculty of Medicine, Charles University and Thomayer Hospital, Prague, Czech Republic; 6 Faculty of Nuclear Sciences and Physical Engineering, Czech Technical University, Prague, Czech Republic; 7 Institute of Neurology, General Hospital and Medical University of Vienna, Vienna, Austria; 8 National Institute of Mental Health, Klecany, Czech Republic; 9 Department of Neurology, Third Faculty of Medicine, Charles University and University Hospital Kralovske Vinohrady, Prague, Czech Republic

**Keywords:** Progressive supranuclear palsy, Atypical parkinsonism, Tauopathies, Spinal cord

## Abstract

**Background:**

We aimed to produce a detailed neuropathological analysis of pyramidal motor system pathology and provide its clinical pathological correlation in cases with definite progressive supranuclear palsy (PSP).

**Methods:**

Pyramidal motor system pathologies were analyzed in 18 cases with neuropathologically confirmed PSP. Based on a retrospective clinical analysis, cases were subtyped according to Movement Disorder Society criteria for clinical diagnosis of PSP as probable, possible or suggestive of PSP with Richardson’s syndrome (*n* = 10), PSP with predominant corticobasal syndrome (*n* = 3), PSP with predominant parkinsonism (*n* = 3), PSP with predominant speech/language disorder (*n* = 1), and PSP with progressive gait freezing (*n* = 1). Clinical manifestations of motor neuron involvement (pseudobulbar or bulbar signs and spasticity) were retrospectively assessed semiquantitatively. Neuropathologically, hyperphosphorylated tau-related pyramidal motor system neuronal, neuritic, and glial pathology using anti-tau AT8 clone immunohistochemistry, was also evaluated.

**Results:**

Clinical manifestations of pyramidal motor system involvement were found in patients with different PSP subtypes. A statistically significant higher load of tau pathology was found in the pyramidal system in PSP-Richardson’s syndrome compared to other PSP subtypes (*p* = 0.016); however, there was no significant correlation between pyramidal system tau pathology and related motor clinical symptoms.

**Conclusions:**

Tau pathology in the spinal cord and pyramidal motor system structures is very common in progressive supranuclear palsy and may neuropathologically supplement the distinction between classic Richardson’s syndrome from other progressive supranuclear palsy subtypes.

**Electronic supplementary material:**

The online version of this article (10.1186/s12883-019-1270-1) contains supplementary material, which is available to authorized users.

## Background

Progressive supranuclear palsy (PSP) is a four-repeat tau predominant (4R-) tauopathy that belongs to the group of frontotemporal lobar degenerations with tau pathology (FTLD-tau) [1].

PSP is the second most common form of parkinsonism after Parkinson’s disease and the third most common geriatric neurodegenerative disorder. PSP prevalence is 18 cases per 100,000 with a median survival from onset to death of approximately 8 years [2, 3].

Typical clinical findings include vertical gaze palsy, gait disturbance, postural instability, frequent falls, akinesia, and symmetric rigidity. Recently, updated diagnostic criteria have been published that clearly describe different clinical PSP presentations [1].

The form with the greatest number of “classic” clinical hallmarks, corresponding to the initial description of PSP by Richardson, Steele, and Olszewski, is now named Richardson’s syndrome (PSP-RS) [4, 5]. Other PSP variants have different clinical presentations, such as initial PSP with predominant parkinsonism (PSP-P: asymmetric dopa-unresponsive parkinsonism with late dementia and falls); PSP with progressive gait freezing (PSP-PGF); PSP with predominant corticobasal syndrome (PSP-CBS: asymmetric dystonia with parkinsonism, limb apraxia, cortical sensory loss, and alien limb); PSP with predominant speech/language disorder (PSP-SL: overlaps the nonfluent/agrammatical variant of progressive aphasia or speech apraxia with parkinsonism); PSP with predominant ocular motor dysfunction (PSP-OM); PSP with postural instability (PSP-PI); PSP with early frontal lobe cognitive or behavioral presentations (PSP-F: including the behavioral variant of frontotemporal dementia with parkinsonism), and very rare forms involving primary lateral sclerosis (PSP-PLS) and cerebellar ataxia (PSP-C) [1].

Involvement of the motor system in PSP thus includes dysfunction at several levels including control of movement by the basal ganglia, brainstem nuclei, and the cerebellum, with appropriate clinical manifestations; upper and lower motor neuron symptomatology is, however, not currently considered to be the dominant clinical feature in the clinical presentation of PSP.

PSP is neuropathologically defined as a 4R-tauopathy with 4R-tau-immunoreactive neuronal, oligodendroglial and astroglial cytoplasmic inclusions, and neuropil threads accompanied by neuronal loss and isomorphic gliosis in the basal ganglia, subthalamic nucleus, substantia nigra, brainstem, cerebellum, and cerebral cortex [6]. Several reports have shown that the spinal cord or the corticospinal tract may be involved in PSP cases [7–15]; however, this is still a rather underappreciated aspect of PSP pathology.

Therefore, in this study, we focused on tau pathology in the spinal cord, corticospinal tract, and primary motor cortex in 18 neuropathologically confirmed PSP cases that presented clinically as either Richardson’s syndrome or as atypical PSP. The aim of our study was (1) to evaluate the extent of tau pathology in the spinal cord and pyramidal motor system in PSP cases and (2) to examine whether the extent of tau pathology in the pyramidal motor system was linked to clinical symptoms of PSP and its subdivision into PSP-RS and PSP-nonRS subtypes.

## Methods

### Cases involved in the study and tissue samples

Written informed consent to participate in the study was obtained from all study participants and/or their next-of-kin/legal guardians. The study protocol was approved by the Multicenter Ethics Committee of the Institute of Clinical and Experimental Medicine and Thomayer Hospital in Prague, Czech Republic (approval nr. G1827).

Brain samples from 18 diseased patients (13 males and 5 females) with definite PSP and without neurodegenerative comorbidity nor serious cerebrovascular pathology were considered for the study.

Their medical records were assessed retrospectively. Basic demographical data are summarized in Table 1 and the Additional file 1: Table S1. All patients underwent a medical autopsy with the subsequent brain and spinal cord autopsy using a standardized protocol involving 3–4 weeks of fixation in 10% neutral buffered formalin. In 4 cases, tissue samples from muscles in the antebrachial and crural regions were also available.

**Table 1:** Basic clinical data of analyzed cases

		n	Gender (M/F)	Age at onset	Duration from disease onset (years)
PSP-RS		10	7/3	62 (51–70)	5.8 (3–9)
PSP-nonRS		8	6/2	70 (60–83)	6 (2–12)
	PSP–CBS	3	2/1	66 (60–76)	6 (2–12)
	PSP-P	3	2/1	68 (66–70)	6.7 (5–7)
	PSP-PGF	1	1/0	83	4
	PSP-SL	1	1/0	77	6
Total		18	13/5	69 (58–83)	5.9 (2–12)

Values (when applicable) are shown as the mean (min–max); PSP subtypes according to the 2017 MDS PSP Criteria (Höglinger et al.)

### Clinical assessment

Data from five clinical centers were included in the study. Medical records of patients with definite PSP were retrospectively analyzed and evaluated by two neurologists (RR, AT). Clinical data are summarized in form of short clinical vignettes as part of the Additional file 2.

First, we analyzed all available data (based on major clinical manifestations at disease onset as well as disease evolution) and assigned, retrospectively, an appropriate PSP subtype to all 18 patients.

Second, we assessed in more detail all available upper and lower motor neuron signs and symptoms. To allow statistical analysis and comparison to neuropathological findings, we created a semiquantitative assessment of pyramidal signs and symptoms (Table 2) in three categories: pseudobulbar signs, bulbar signs, and spasticity.

**Table 2:**
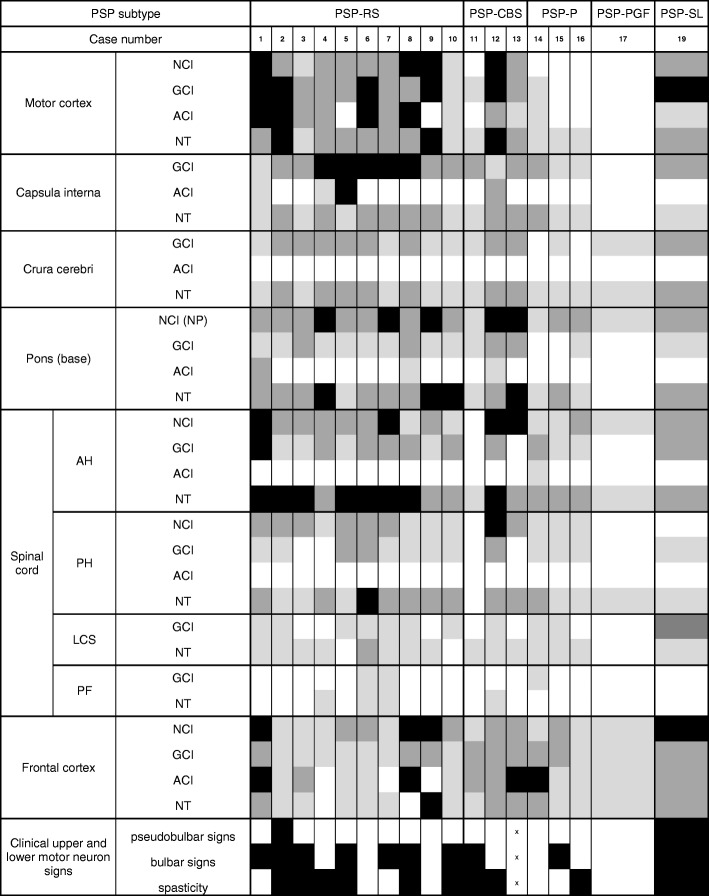
Clinical and AT8-tau pathology scoring for specific brain and spinal cord areas (domains). PSP progressive supranuclear palsy; PSP-RS PSP-Richardson syndrome; PSP-CBS PSP-corticobasal syndrome; PSP-P PSP-parkinsonism; PSP-PGF PSP pure akinesia with progressive gait freezing; PSP-SL PSP speech/language disorder; NCI neuronal cytoplasmic inclusions; NP nuclei pontis; GCI oligodendroglial cytoplasmic inclusions; ACI astroglial cytoplasmic inclusions; NT neuropil threads and dots; ANT anterior spinal nerve root; AH spinal cord anterior horn; LCS: lateral corticospinal tract; PH spinal cord posterior horn; PF posterior fascicles

Colours indicate the load of respective tau pathology: black = severe (3); dark grey = moderate (2); light grey = mild (1); white = absent (0). Colours in the clinical rows indicate presence or absence of given signs: black = present (1); white = absent (0); x = data not available

Bulbar signs were rated as 0 – absent, 1 – manifest (paralytic dysarthria, severe dysphonia, dysphagia, and/or a weak cough and susceptibility to aspiration).

Pseudobulbar signs were rated as 0 – absent, 1 – manifest (spastic dysarthria, labile emotions and/or unprovoked outbursts of laughing or crying, or the previous manifestations together with frontal gait apraxia).

Spasticity signs were rated as follows: 0 – absent, 1 – manifest (evident hyperreflexia with Babinski sign, and/or a marked increase in muscle tone).

### Immunohistochemistry and histochemistry

Paraffin-embedded tissue sections (4 μm) from different regions (Table 2) were examined using routine hematoxylin-eosin and luxol-eosin stains. Immunohistochemistry was performed using antibodies against hyperphosphorylated tau protein (clone AT8, 1:500, Thermo Scientific), 4R-tau isoform (RD4, clone 1E1/AG, 1:200, Upstate), 3R-tau isoform (RD3, clone 8E6/C11, 1:500, Upstate), p62 (clone GP62, 1:4000, PROGEN), and ubiquitin (polyclonal, 1:500, Dako) with low-pH citrate buffer epitope retrieval. Moreover, antibodies against amyloid-beta (clone 6F/3D, 1:100, Dako), alpha-synuclein (4D6, 1:10000, Signet), and phospho-TDP-43 (clone 11/9, 1:4000, CosmoBio), were used in the context of a differential diagnostic workup. Subsequent visualization of all antibodies was performed using a horseradish peroxidase–diaminobenzidine system (Envision FLEX/HRP, polyclonal rabbit-anti-guinea pig; Dako).

### Semiquantitative assessment of tau pathology

All morphometric scoring was performed independently by two neuropathologists (ZS, ZR). Analyzed areas in all PSP cases (*n* = 18) included the primary motor cortex, frontal cortex, internal capsule, basal ganglia (striatum), cerebral peduncles, pons base, cerebellum (area of the dentate nucleus). Spinal cord (anterior and posterior horn and lateral corticospinal tract), and the anterior and posterior spinal nerves (posterior fascicles and radices) were also evaluated (see Table 2).

**Fig. 1:**
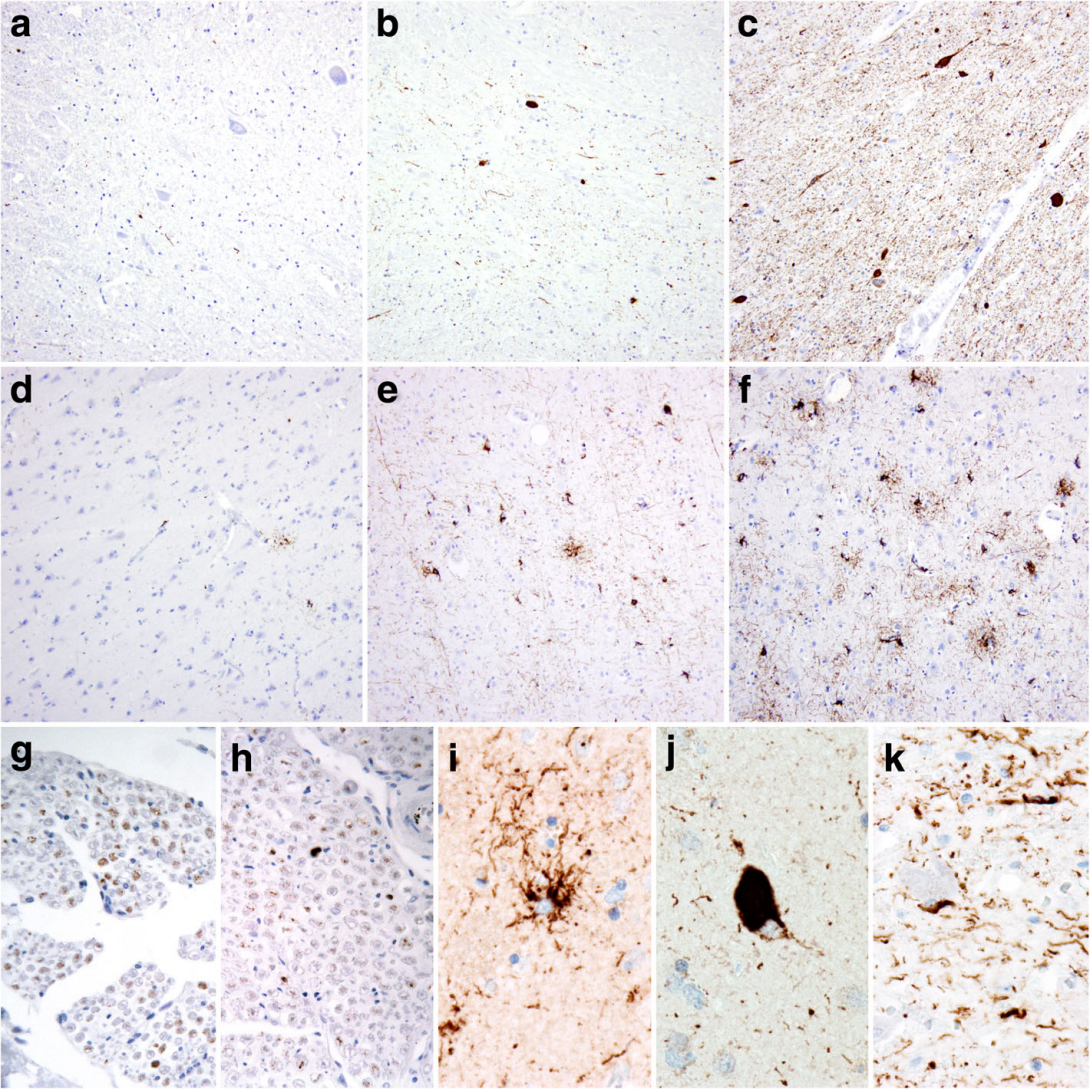
Examples of neuropathological changes in the examined PSP cases. Spinal cord (**a**–**c**) and primary motor cortex (**d**–**f**) tau (AT8 antibody) scoring: **a**, **d** + (mild); **b**, **e** ++ (moderate); **c**, **f** +++ (severe); g, h tau-AT8-immunoreactivity in the anterior roots of a spinal nerve; 4R-tau (RD4 antibody)-immunoreactive tufted astrocyte (**i**), globose neurofibrillary tangle; **j** and oligodendroglial coiled inclusions; **k** with neuropil threads and dots in the background. Magnification **a**–**f** 200x; **g**–**k** 400x

We defined PSP-related AT8-tau immunoreactive (AT8-ir) structures as AT8-ir globose and neurofibrillary tangles (NFT)-like inclusions (NCIs), oligodendroglial coiled-body-type cytoplasmic inclusions (GCIs), astroglial cytoplasmic inclusions (tufted astrocytes; TAs), and neuropil AT8-ir threads and dots (NTs). Semi-quantification of AT8-tau pathology was assessed as absent (0), mild (1), moderate (2), and severe (3) (Fig. 1).

None of the cases included in the study showed motor neuron disease/amyotrophic lateral sclerosis-associated pathology in the spinal cord or motor cortex including significant neuronal loss and Bunina bodies in the spinal cord anterior horn motor neurons, demyelination of the lateral and anterior corticospinal tracts or phosphorylated TDP-43 or p62- and ubiquitin-related associated inclusion pathology. Other types of neuronal, glial, and neuropil pathology were recorded separately, mainly as concomitant Alzheimer disease, Lewy body disease, or neuronal and neuropil tau pathology in the form of primary age-related tauopathy (PART), or as astroglial tau pathology that presented as recently described age-related tau astrogliopathy (ARTAG) [16]. None of the cases met the morphologic criteria for globular glial tauopathy (GGT) [17].

### Statistical methods

The statistical analysis focused on specific brain and spinal cord areas (domains) defined neuropathologically (motor cortex, capsula interna, crura cerebri, pons, anterior spinal horns, posterior spinal horns, lateral corticospinal tract, posterior fascicles) and their neurodegenerative changes.

Using the two-sample two-sided Wilcoxon-Mann-Whitney (WMW) rank-sum test on groups of PSP-RS and PSP-nonRS patients, we verified the H0 hypothesis (PSP-RS versus PSP-nonRS patients) regarding the equality of score medians at a critical level of *p* = 0.05.

We tested both individual domain score sums and the total domain score. The same WMW test at *p* = 0.05 was used to score clinical pyramidal syndrome in PSP-RS vs. PSP-nonRS patients.

The relationship between neuropathological scores and clinical pyramidal signs was tested using the Spearman’s test (level of significance *p* = 0.05).

## Results

Clinical scoring and AT8-tau pathology of all 18 analyzed cases (including those not statistically evaluated) are summarized in Table 2.

### Clinical manifestation

The principal clinical findings are reported in detail as clinical vignettes provided as Additional file 2: Text.

The most frequent PSP subtype, Richardson’s syndrome, was present in 10 patients. Eight cases had visible bulbar and pseudobulbar manifestations and 6 had spasticity signs.

In the PSP-nonRS group, bulbar and pseudobulbar syndromes were found in one PSP-CBS, and one PSP-P. Spasticity manifestation included two PSP-CBS, one PSP-P, and one PSP-SL patient (Table 2).

In analyzing clinical scores of the patients, we found no significant difference between the RS and non-RS subgroups relative to their local clinical scores.

### Spinal cord and motor cortex AT8-tau pathology were present in the majority of PSP cases

All 18 cases had at least one segment of the spinal cord (cervical or lumbar) available for evaluation. In these cases, AT8-tau pathology, from a mild (1) to a severe (3) degree, affected the spinal gray matter in the anterior horns as well as the primary motor cortex (M1) (Table 2), which clearly showed involvement of the upper as well as lower motor neuron systems. The morphological spectrum of spinal cord AT8-ir structures mainly included NTs and, to a lesser extent, AT8-ir GCIs and NCIs in the anterior horns. AT8-ir GCIs and NTs were visible in the lateral corticospinal tracts in 17 cases, and in 5 cases we observed rare AT8-ir in the posterior fascicles. Astrocytic AT8-ir structures in the anterior horn, in the form of TAs, were only observed in one case. Moreover, in 7 cases we found AT8-ir NTs in the anterior roots of spinal nerves and 6 cases had AT8-ir threads in the posterior roots. In all cases, the 4R-tau isoform dominated, as visualized using the RD4 antibody (Fig. 1). Routine luxol-eosin staining did not show significant degeneration of corticospinal tracts.

No p62- or AT8-ir inclusions were found in samples from skeletal muscles, which included peripheral nerve branches as well as striated muscle tissue.

In the primary motor cortex, AT8-ir, 4R-tau predominant TAs, GCIs, NCIs, and NTs were present in the gray matter and at the cortico-subcortical interface. Moreover, adjacent parts of the primary sensory cortex showed a lower tau pathology load than did the primary motor cortex (M1). Tau pathology in the superior frontal gyrus was more variable and did not consistently correspond to the tau pathology seen in M1 (Table 2).

### Load of AT8-related tau pathology in the motor system distinguishes PSP-RS from non-RS

Applying the WMW test to the total neuropathological score of the motor cortex, internal capsule, midbrain, anterior horn, posterior horn, lateral corticospinal, and posterior fascicles, we observed a statistically significant difference between RS (median = 36, mean = 34.8, min = 25, max = 43) and non-RS (median = 22, mean = 23.4, min = 6, max = 45) groups with a *p*-value = 0.016.

There were no statistically significant differences between PSP-RS and non-RS patients in any of the previously mentioned individual brain areas.

### AT8-tau pathology was variably present at all examined levels of the corticospinal tract

Even though significantly higher in PSP-RS than PSP-nonRS cases, AT8-tau pathology was detectable in all cases in the corticospinal tract including the internal capsule, cerebral peduncles, and pontine fibers, mainly in the form of AT8-ir NTs and AT8-ir GCIs (Table 2).

In PSP-RS cases, the AT8-related tau pathology load of M1 was in line with the scoring of tau pathology by Williams et al. [18]. In non-RS subtypes, M1 was inconsistently affected, with the lowest amount of AT8-tau pathology in PSP-P and PSP-PGF cases (Table 2).

## Discussion

We present a systematic clinical and neuropathological analysis of the pyramidal motor system and spinal cord tau pathology in 18 neuropathologically confirmed PSP cases, including different PSP variants: PSP-RS, PSP-CBS, PSP-P, PSP-PGF, and PSP-SL.

In our cohort, we analyzed and statistically compared both neuropathological and clinical data. Important concerns, however, were the very heterogeneous clinical descriptions coming from the multiple centers involved and the completely retrospective nature of our study. We addressed these concerns by using neuropathological analyses that were done according to the same protocols in one center, which created morphologic data which were homogeneous across all cases. To counterbalance this inconvenience and to analyze clinical data statistically, we developed our own semiquantitative assessment system since standardized clinical rating scales could not be applied retrospectively in all cases because of inconsistencies in clinical descriptions from different centers.

PSP can be broadly viewed as a multisystem neurodegenerative disorder due to its propensity to involve various, mainly motor control-associated brain areas. This is apparent from both clinical and neuropathological standpoints. From the clinician’s view, PSP has, for a quite some time, been considered a homogenous disease associated with poor dopa-responsive parkinsonism, subcortical dementia, gait disturbance, early and frequent falls, incontinence, and supranuclear gaze palsy. However, there is increasing evidence of far more heterogeneity in PSP presentation, both clinically and neuropathologically [1, 19], than previously thought.

Spinal cord pathology in PSP, with neuronal loss and gliosis in the anterior horns, was first described in 1974 by Ishino et al. [7]. Several subsequent studies confirmed the presence of NFTs in spinal cord grey matter (using silver impregnation methods, electron microscopy, and anti-tau antibodies); however, evidence regarding their clinical relevance is still lacking [8–12, 14, 15, 20].

In a recent study of 3 typical and atypical PSP cases, Zhu et al. reported two cases of globose NFTs, NTs, and TAs in the anterior horns and surrounding white matter of the cervical spinal cord, although, they did not focus on the motor system [13].

Regarding corticospinal tract pathology, Josephs et al. described 12 atypical PSP cases with involvement of the internal capsule. However, these cases were subsequently reclassified as globular glial tauopathy [17, 21]. The question of whether clinical observations of patients with features of PSP and PLS [1] are associated with the presence of globular oligodendroglial inclusions in the corticospinal tract [21] merits further investigation.

Williams et al. suggested that RS (Richardson’s syndrome corresponding to the “classic” form of PSP) differed from PSP subtypes having a more focal involvement (i.e., dopa-responsive parkinsonism and frequent absence of gaze palsy in PSP-P, predominately gait disturbance in PSP-PAGF, early and pronounced parietal lobe impairment in PSP-CBS, or progressive aphasia in PSP-SL) in both disease severity, rapidity of progression, and extent of neuropathological changes [18]. These findings are in line with our neuropathological observation of widespread tau deposits in pyramidal structures (in all PSP cases) and a significantly greater involvement in PSP-RS compared to non-RS forms. Surprisingly, the clinical evidence was far less convincing.

In one case (case 2, see Additional file 2: Text) the patient progressed as expected with a typical form of RS, but later in the disease course signs of motor neuron disease developed (severe pseudobulbar and bulbar syndrome with hypersalivation, dysarthria evolving towards virtual mutism, dysphagia, a paralyzed and atrophic tongue, emotional incontinence, and hyperreflexia), which led us to consider comorbid motor neuron disease, although the EMG diagnostic criteria were not met. Similar cases have been reported in the literature [22].

Another case (case 16, see Additional file 2: Text) developed parkinsonism with akinesia and rigidity, which initially responded to levodopa. However, the disease progressed rapidly, and the patient became unable to walk within two years due to lower body parkinsonism, marked gonarthrosis, and spasticity of the lower extremities. Similarly, Papapetropoulos et al. found PSP-like diffuse tangle involvement in the spinal cord of a patient with a gradually progressive spastic gait, late supranuclear gaze palsy, and cognitive impairment [15].

As discussed before, the results of our study are in line with previous studies describing tau pathology in the spinal cord. However, the novelty of our study is linked to its more systematic analysis of the different levels of corticospinal tract involvement and correlations with clinical data. Interestingly, except for one case, none of the PSP cases presented with clinically significant upper or lower motor neuron symptomatology, despite moderate to severe tau pathology in many cases. The most common tau pathologies in the anterior horns were AT8-ir NCIs, NTs, and, to a lesser extent, GCIs; TAs were observed only in one PSP-P case. In view of these features as well as reports from the literature [21], it might be theorized that prominent corticospinal tract degeneration with corresponding clinical symptomatology are associated with massive oligodendroglial inclusions (globular inclusions) instead of coiled bodies as seen in our cases, which supports the concept that globular inclusions and not coiled bodies are markers of tract degeneration [23].

Since astroglial spinal cord AT8-tau pathology, as well as typical peripheral motor neuron symptomatology, were largely absent from both the upper and lower extremities in our cases, we can speculate that in PSP, glial dysfunction, rather than NTs or NCIs, in other areas of the pyramidal tract may be primarily responsible for PSP pathogenesis and clinical symptomatology. Indeed, it was shown that astroglial tau pathology frequently precedes neuronal tau pathology in clinically relevant anatomical regions in cases of primary FTLD-tauopathies, suggesting that it is an indicator of early involvement of the degenerative process [24, 25].

Our study has two main limitations: first, even though large enough to allow for valid statistical analysis, the number of studied cases was limited, especially for certain subtypes of non-RS PSP cases, and second, the clinical analysis used in our study was based on a retrospective evaluation of medical records from five different centers. Therefore, a prospective study with a larger cohort of neuropathologically confirmed PSP cases would provide more precise conclusions regarding pyramidal motor system pathology in PSP cases.

## Conclusions

The main findings of our study are in line with certain previously published neuropathological observations. They demonstrate that pyramidal cells and the pyramidal tract are involved in PSP patients and that the PSP-RS forms are significantly more affected than non-RS forms. We added to these findings clinical insight showing the occurrence of pyramidal signs in many PSP patients. However, our study failed to find any similar significant differences between RS and non-RS forms relative to clinical symptomatology.

Since PSP-RS patients frequently have faster disease progression and greater disability than non-RS forms, and since individual prognostic markers for PSP patients are still lacking, the detection of pyramidal symptoms in PSP may have prognostic importance, although our study was not designed primarily to address this issue.

Further studies are needed to ascertain the full value of a targeted clinical assessment of pyramidal signs in PSP patients.

## Additional files

Additional file 1: **Table S1.** Detailed demographic and other description of individual cases. (DOCX 19 kb)

Additional file 2: Individual case clinical description. (DOCX 32 kb)

## Abbreviations

3R: Three repeat tau isoform
4R: Four repeat tau isoform
ALS: Amyotrophic lateral sclerosis
ARTAG: Age-related tau astrogliopathy
FTLD-tau: Frontotemporal lobar degenerations with tau pathology
GCI: Oligodendroglial coiled-body-type cytoplasmic inclusions
GGT: Globular glial tauopathy
ir: Immunoreactivity
LCS: Lateral corticospinal tract
M1: Primary motor cortex
NCI: Neuronal cytoplasmic inclusions
NFT: Neurofibrillary tangles
NTs: neuropil threads and dots
PART: primary age-related tauopathy
PSP: progressive supranuclear palsy
PSP-C: PSP with predominant cerebellar ataxia
PSP-CBS: PSP-corticobasal syndrome
PSP-F: PSP with early frontal lobe cognitive or behavioral presentations
PSP-nonRS: PSP other subtypes than Richardson’s syndrome
PSP-OM: PSP with predominant ocular motor dysfunction
PSP-P: PSP with parkinsonism
PSP-PGF: PSP-pure akinesia with progressive gait freezing
PSP-PI: PSP with postural instability
PSP-PLS: PSP with features of primary lateral sclerosis
PSP-RS: Richardson’s syndrome
PSP-SL: PSP-speech/language disorder
TAs: astroglial cytoplasmic inclusions
WMW: Wilcoxon-Mann-Whitney test
