# Supplementary material for: Pyramidal system involvement in progressive supranuclear palsy – a clinicopathological correlation
Source: BMC Neurol. 2019 Mar 20;19:42. doi: 10.1186/s12883-019-1270-1 (PMC6425568; doi:10.1186/s12883-019-1270-1)
Supplement: Supplementary file 1 — Table S1. Detailed demographic and other description of individual cases. (DOCX 19 kb) [file 12883_2019_1270_MOESM1_ESM.docx]

|  | **Gender** | **Age at disease onset** | **Approximative disease duration [months]** | **Brain weight [g]** | **PMD (h)** | **Cause of death** |
| --- | --- | --- | --- | --- | --- | --- |
| **1** | M | 70 | 48 | 1400 | 23 | BP |
| **2** | F | 68 | 72 | 1180 | 55 | BP |
| **3** | M | 68 | 48 | 1270 | 24 | BP |
| **4** | F | 59 | 48 | 1270 | 48 | BP |
| **5** | M | 57 | 72 | 1560 | 23 | CRF |
| **6** | M | 57 | 108 | 1220 | 43 | CF |
| **7** | M | 55 | 36 | 1350 | 80 | BP |
| **8** | F | 63 | 96 | 1000 | 183 | BP |
| **9** | M | 57 | 60 | 1420 | 20 | BP |
| **10** | M | 49 | 108 | 1610 | 16 | CF |
| **11** | M | 53 | 84 | 1190 | 48 | BP |
| **12** | M | 60 | 60 | 1300 | 18 | BP |
| **13** | F | NA^*^ | NA | 1166 | NA | BP |
| **14** | F | 66 | 24 | 1200 | 23 | CF |
| **15** | M | 56 | 144 | 1270 | 68 | BP |
| **16** | M | 60 | 72 | 1360 | 13 | CF |
| **17** | M | 79 | 48 | 1150 | 45 | CF |
| **18** | M | 77 | 72 | 1290 | 64 | BP |

**Table S1 Cases included in the study. PMD** post mortem delay; **BP** bronchopneumonia; **CRF** chronic renal failure; **CF** cardiac failure; ^*^ age at death 76 years; **NA** not available
