# Supplementary material for: Pyramidal system involvement in progressive supranuclear palsy – a clinicopathological correlation
Source: BMC Neurol. 2019 Mar 20;19:42. doi: 10.1186/s12883-019-1270-1 (PMC6425568; doi:10.1186/s12883-019-1270-1)
Supplement: Supplementary file 2 — Individual case clinical description. (DOCX 32 kb) [file 12883_2019_1270_MOESM2_ESM.docx]

## Case 1

A 70-year-old man developed progressive gait instability and blurred vision. Neurological examination showed subcortical dementia, rigidity with akinesia, but without tremor, supranuclear gaze palsy with limitation of both horizontal and vertical eye movements, dysarthria, limb ataxia, and unstable gait. CT scan was normal. Progressively, gait apraxia and parkinsonism worsened and occasional falls occurred, while the oculomotor palsy became a serious impediment to daily living. The patient was diagnosed as having probable PSP with Richardson’s syndrome (prob. **PSP-RS**) and died of bronchopneumonia 4 years after disease onset.

## Case 2

A 70-year-old woman presented with a two-year history of blurred vision, gait instability, psychomotor slowing, and apathy. On assessment, predominant objective signs included hyperreflexia, axial rigidity and akinesia, vertical gaze palsy with gaze apraxia, and behavior perseverations, and echopraxia. MRI showed mild mesencephalic and frontal atrophy; neuropsychological testing found noticeable psychomotor slowing, executive dysfunction, and considerable attention impairment. The condition rapidly worsened and over three years she developed pseudobulbar and bulbar signs, mainly in the form of severe paralytic dysarthria and dysphagia, suggesting possible motor neuron disease and, percutaneous endoscopic gastrostomy (PEG) was introduced. Muscle strength in the extremities was preserved, but frequent falls occurred with multiple injuries; worsening postural instability and gait apraxia made active locomotion almost impossible. The patient died from bronchopneumonia 6 years after the first manifestations. Probable PSP with Richardson’s syndrome (prob. **PSP-RS**) was the clinical diagnosis.

## Case 3

A 68-year-old man slowly developed memory disturbances, gait instability, and occasional falls. Neurological examination found slow oculomotor saccades, apathy, frontal inhibition, and hypokinesia, but no rigidity. MRI showed symmetric temporal and hippocampal atrophy associated with subcortical white matter lesions (Fazekas stage 2). A neuropsychological assessment revealed significant episodic memory impairment with encoding and retrieval difficulties and confabulations together with considerable executive dysfunction. The initial clinical diagnosis was frontal variant of Alzheimer’s disease, however, despite treatment with acetylcholinesterase inhibitors dementia progressed. DaTSCAN was positive and the disease worsened rapidly to akinesia with rigidity, severe subcortical dementia, gaze apraxia and vertical gaze limitation, corresponded to probable PSP with Richardson’s syndrome (prob. **PSP-RS**). Ultimately, the patient was unable to walk and died from bronchopneumonia 4 years after the initial onset.

## Case 4

A 60-year-old woman presented with memory loss and attention deficits. Severe subcortical dementia developed progressively. MRI showed atrophy with frontotemporal predominance. Neuropsychological assessment demonstrated found impaired episodic memory with confabulations and executive dysfunction, however, visuospatial abilities were basically preserved. Two years later, her condition worsened, with depression, massive behavior perseverations, stereotypia, significant reduction in verbal fluency, and considerable grasping. Neurological examination showed akinesia and rigidity with an axial prevalence. Supranuclear gaze palsy and postural instability with gait apraxia and frequent falls were late features. Initially, Alzheimer´s disease was suspected; however, as the disease progressed, probable PSP with Richardson´s syndrome (prob. **PSP-RS**) was considered the adequate diagnosis. The patient died from terminal bronchopneumonia four years after the first clinical symptoms appeared.

## Case 5

A 60-year-old man presented with a three-year history of gait instability and frequent falls. Clinical examination showed psychomotor slowing, apathy, inconstant irritability, dysarthria, gait apraxia, manifest limb ataxia, and mild akinesia with rigidity. MRI found posterior pontine and mesencephalic atrophy. DaTSCAN was positive and a neuropsychological assessment found predominant attention and executive dysfunction and impaired retrieval on episodic memory tests. The clinical diagnosis at this time was multiple system atrophy. Progressively, akinesia worsened, vertical supranuclear gaze palsy developed, number of falls increased, and the patient became demented. MRI showed progression of the mesencephalic atrophy leading us to conclude to probable PSP with Richardson’s syndrome (prob. **PSP-RS**). The patient died of chronic renal failure 6 years after the disease first manifested.

## Case 6

A 66-year-old man with a history of akinesia, rigidity, and frontal lobe dementia with severe bradypsychia and frequent repeated falls subsequently developed early vertical gaze limitation and was diagnosed with probable PSP with Richardson’s syndrome (prob. **PSP-RS**). He died 9 years after disease onset from sudden circulatory arrest in the context of depression and malnutrition.

## Case 7

A 58-year-old man developed parkinsonism with dementia. He was examined several months later in a movement disorders center. Since he presented with levodopa resistant akinesia, rigidity and postural instability, subcortical dementia, and impaired downward gaze palsy, he was diagnosed with possible PSP with Richardson’s syndrome (poss. **PSP-RS**). Progressively he developed severe bulbar syndrome with dysarthria and dysphagia and died from pneumonia about three years after disease onset.

## Case 8

A 63-year-old woman developed a gait disturbance with frequent falls, associated with dementia and slowed vertical gaze saccades. Because of symmetric akinesia and rigidity, levodopa was introduced, up to 1 g daily, however, without noticeable effect. The condition rapidly deteriorated, and after four years, she was severely demented, almost mute, with massive echopraxia, nearly complete voluntary supranuclear gaze palsy (but preserved horizontal pursuit), axial dystonia, severe akinesia with proximal prevalence, and incontinence. MRI showed atrophy of the dorsal midbrain. She died 8-years after onset of probable PSP with Richardson’s syndrome (prob. **PSP-RS**), from terminal bronchopneumonia.

## Case 9

A 59-year-old man presented with a 2-year history of progressive amnestic dementia and apraxia. Cognitive assessment detected loss of episodic memory with intrusions, visuospatial and executive impairment, and anosognosia. Two years later, his executive functions markedly deteriorated, with prominent apathy, stereotypia, perseverations, and parkinsonism features develop, (e.g., symmetric axial rigidity and hypomimia (with only mild akinesia and inconstant resting tremor). Levodopa was given at a daily dose of 1.5 g, however, without improvement. One year later, the development of a downward gaze palsy with frequent falls, orthostatic hypotension, incontinence, dysphagia, and frontal lobe dementia was strongly suggestive of probable PSP with Richardson´s syndrome (prob. **PSP-RS**). MRI detected midbrain and frontotemporal atrophy. The parkinsonism and dementia was progressive; later he developed dressing apraxia, nonfluent aphasia, and occasional visual hallucinations. He died from terminal bronchopneumonia five years after disease onset.

## Case 10

A 49-year-old patient complained of low back pain while walking, erectile dysfunction, and hypersalivation. Over the next four years he developed dysarthria, dysphagia, and an unstable gait with occasional falls. Neurological examination showed frontal disinhibition, palsy of vertical eye movements, postural instability, and gait apraxia. Brain MRI revealed only unspecific small subcortical vascular white matter lesions. During the following five years his status progressively deteriorated, which led to severe dementia and parkinsonism. He died of cardiac failure nine-years after the onset of what strongly appeared to be probable PSP with Richardson’s syndrome (prob. **PSP-RS**).

## Case 11

A 53-year-old man developed ideomotor apraxia with alien-limb phenomenon on the left side. MRI showed considerable atrophy mainly in the right central and parietal regions. Akinesia and rigidity without tremor remained strikingly asymmetric and spasticity progressed on the right upper extremity. Neuropsychological examination demonstrated executive dysfunction, significant left-sided apraxia, and nonfluent aphasia with agrammatism, paraphasias, aprosody, and impaired repetition. Supranuclear gaze palsy was a very late finding. The patient died from terminal bronchopneumonia seven years after the first manifestations of possible PSP with predominant corticobasal syndrome (poss. **PSP-CBS**).

## Case 12

A 61-year-old man had a 16-month history of dementia with early falls and movement disorders. Clinical examination found rigidity and akinesia (without tremor), mainly on the left side with an alien-hand phenomenon, and apraxia. The patient was generally apathetic, however, with irritability and episodes of reactive verbal aggressiveness. Oculomotor saccades were less than expected and vertical gaze was considerably restrained. Gait was apraxic and unstable; multiple injuries from various falls at different ages were visible on the skin. Neuropsychological examination found predominant visuospatial impairment and executive dysfunction (decreased verbal fluency, abstract thinking and reasoning, extensive behavior perseverations); MRI showed frontal, parietal, and mesencephalic atrophy. His condition deteriorated progressively to severe dementia with rigidity (patient became bedridden) and he died from bronchopneumonia 5 years after the first manifestations of the disease. Possible PSP with predominant corticobasal syndrome (poss. **PSP-CBS**) was the most likely clinical diagnosis.

## Case 13

A 76-year-old woman developed atypical parkinsonism with strictly unilateral right-sided akineto-rigid syndrome, severe dysphagia, and executive dysfunction with reduced verbal fluency progressing to mutism. The clinical diagnosis was retrospectively considered suggestive of PSP with predominant corticobasal syndrome (s.o. **PSP-CBS**). Because of the dysphagia, PEG was introduced, despite this, the patient died a few days later from septic bronchopneumonia.

## Case 14

A 66-year-old woman was referred to a neurosurgeon with normal pressure hydrocephalus (gait instability, incontinence, frequent falls, and apathy). MRI showed cortical and subcortical atrophy and ventricular dilatation. However, even after ventriculoperitoneal shunting, the clinical manifestation progressively worsened. Neurological examination at this time found a resting tremor that was more prevalent on the left side, akinesia and rigidity, severe frontal gait apraxia, and executive dysfunction (perseverations and stereotypy, decreased verbal fluency and initiation, apathy, emotional inhibition, and imitation apraxia). The clinical presentation strongly suggested Parkinson’s disease or retrospectively suggestive of PSP with predomiant parkinsonism **(s.o. PSP-P).** Treatment with levodopa was started, however, the patient died suddenly, from a heart attack, 2 years after disease onset.

## Case 15

A 64-year-old man was followed, for 8 years, by a local neurologist, with a diagnosis of Parkinson’s disease with an akineto-rigid syndrome and questionable dopa-sensitivity, which led to a referral to a specialized center. The dose of levodopa was increased, but his condition rapidly deteriorated with development of severe frontal lobe dementia, frequent falls, incontinence, and a fixed dystonic posture of the neck. Vertical gaze palsy was a very late feature. He died from bronchopneumonia 12 years after the first manifestation of disease. The clinical presentation strongly suggested probable PSP with predominant parkinsonism **(prob. PSP-P)**.

## Case 16

A 60-year-old man developed parkinsonism with akinesia and rigidity, but no tremor, initially he responded to high dose levodopa (1.5 g daily). His gait was difficult to analyze because of severe bilateral gonarthrosis and bilateral crural ulcerations, however, surgery was ruled-out because of severe ischemic heart disease and occlusive arterial disease in his lower extremities. The disease progressed very slowly, cognitive function remained preserved with only mild executive dysfunction; oculomotor abnormalities developed late in the disease course. Brain MRI was normal. The patient died from sudden cardiac arrest 6 years after disease onset. This presentation corresponded to probable PSP with predominant parkinsonism **(prob. PSP-P**).

## Case 17

An 81-year-old man presented with a two-year history of progressive gait instability, occasional falls, and psychomotor slowing, which appeared to be associated with forgetfulness (mainly late recall difficulties), partial anosognosia, social withdrawal, and dressing neglect. On clinical assessment, severe gait apraxia (with instability) and difficulty initiating walking were the predominant features, as well difficulty standing up after a fall. Cognitive impairment predominated on memory and executive functions tests (set shifting, abstract thinking, judgment, emotional control, initiative). Oculomotor movements were not affected and a CT scan was normal. He died four years after disease onset from cardiac failure. This presentation was suggestive of possible PSP with progressive gait freezing **(poss. PSP-PGF**).

## Case 18

A 77-year-old man progressively consulted a memory clinic for impaired ability to speak, decreased verbal fluency (with preserved comprehension and repetition); additionally, there were considerable word finding difficulties, dysarthria, and levodopa unresponsive parkinsonism. Two years later, vertical gaze was severely restricted (horizontal gaze to a lesser degree) and the patient became incontinent. He died 6 years after disease onset from terminal pneumonia. This evolution was compatible with possible PSP with predominant speech/language disorder **(poss. PSP-SL**).
